# HELP LDL Apheresis Reduces Plasma Pentraxin 3 in Familial Hypercholesterolemia

**DOI:** 10.1371/journal.pone.0101290

**Published:** 2014-07-11

**Authors:** Michela Zanetti, Mariagrazia Zenti, Rocco Barazzoni, Federica Zardi, Annamaria Semolic, Michele Giuseppe Messa, Filippo Mearelli, Gianpaolo Russi, Maurizio Fonda, Luca Scarano, Enzo Bonora, Luigi Cattin

**Affiliations:** 1 Clinica Medica, Department of Medical, Surgical and Health Sciences, University of Trieste, Trieste, Italy; 2 Endocrinology, Diabetes and Metabolism, University and Azienda Ospedaliera Universitaria Integrata of Verona, Verona, Italy; 3 Nephrology, University and Azienda Ospedaliera Universitaria Integrata of Verona, Verona, Italy; 4 Diabetes and Metabolic Diseases Unit, Department of Medical, Surgical and Health Sciences, University of Trieste, Trieste, Italy; 5 Immunohematology and Transfusion Unit, Ospedale Santa Maria Nuova, Reggio Emilia, Italy; Maastricht University, Netherlands

## Abstract

**Background:**

Pentraxin 3 (PTX3), a key component of the humoral arm of innate immunity, is secreted by vascular cells in response to injury, possibly aiming at tuning arterial activation associated with vascular damage. Severe hypercholesterolemia as in familial hypercholesterolemia (FH) promotes vascular inflammation and atherosclerosis; low-density lipoprotein (LDL) apheresis is currently the treatment of choice to reduce plasma lipids in FH. HELP LDL apheresis affects pro- and antiinflammatory biomarkers, however its effects on PTX3 levels are unknown. We assessed the impact of FH and of LDL removal by HELP apheresis on PTX3.

**Methods:**

Plasma lipids, PTX3, and CRP were measured in 19 patients with FH undergoing chronic HELP LDL apheresis before and after treatment and in 20 control subjects. In the patients assessment of inflammation and oxidative stress markers included also plasma TNFα, fibrinogen and TBARS.

**Results:**

At baseline, FH patients had higher (p = 0.0002) plasma PTX3 than matched control subjects. In FH PTX3 correlated positively (p≤0.05) with age, gender and CRP and negatively (p = 0.01) with HELP LDL apheresis vintage. The latter association was confirmed after correction for age, gender and CRP. HELP LDL apheresis acutely reduced (p≤0.04) plasma PTX3, CRP, fibrinogen, TBARS and lipids, but not TNFα. No association was observed between mean decrease in PTX3 and in LDL cholesterol. PTX3 paralleled lipids, oxidative stress and inflammation markers in time-course study.

**Conclusion:**

FH is associated with increased plasma PTX3, which is acutely reduced by HELP LDL apheresis independently of LDL cholesterol, as reflected by the lack of association between change in PTX3 and in LDL levels. These results, together with the finding of a negative relationship between PTX3 and duration of treatment suggest that HELP LDL apheresis may influence both acutely and chronically cardiovascular outcomes in FH by modulating PTX3.

## Introduction

A body of clinical and experimental evidence supports a link between LDL cholesterol, inflammation and atherosclerosis [1]–[3]. Cholesterol accumulation in the artery triggers a local and systemic inflammatory reaction by activating multiple pathways, including native and adaptive immune responses [4]–[5]. Pentraxin 3 (PTX3), a component of the humoral arm of native immunity, is a TNFα-inducible molecule selectively produced following activation of the vessel wall by several cell types at the site of injury [6]–[8]. PTX3 is strictly associated with vascular disease, as high levels have been detected in atherosclerotic plaques and in plasma from patients with elevated LDL cholesterol and extensive atherosclerosis [9]–[11]. In hypercholesterolemia PTX3 correlates with the severity of vascular disease and statin therapy reduces plasma PTX3 [11], suggesting its involvement in the mechanisms by which LDL cholesterol triggers vascular inflammation. So far, the precise role of PTX3 in atherosclerosis is unclear. The finding that PTX3 deficiency amplifies tissue damage and inflammation after vascular injury [12] suggests a protective activity, which however needs to be proven in human studies.

LDL apheresis is the treatment of choice for severe hypercholesterolemia not adequatelly controlled by maximal statin treatment, as in familial hypercholesterolemia (FH) [13]–[14]. Patients affected by FH undergoing LDL apheresis are a paradigmatic model since LDL cholesterol rapidly drops from elevated to normal levels and then slowly rebounds to pre-apheresis concentrations [15]. In addition to LDL cholesterol, LDL apheresis acutely reduces inflammation and oxidative stress markers, including pro-inflammatory cytokines, adhesion molecules, oxidative products as well as coronary endothelial dysfunction [16]–[19]; therefore positive effects overwhelm possible detrimental consequences of endothelial activation following blood-biomaterial interaction during the procedure. For these reasons LDL apheresis has been proposed in the acute treatment of acute coronary syndromes (ACS) [20]. Chronic LDL apheresis delays the progression of atherosclerosis, stabilizes atherosclerotic plaques and reduces clinical events in FH [21]–[22]. These beneficial effects are paralleled by long-term reduction in several inflammatory biomarkers including CRP, which is notably associated with clinical endpoints [23]–[24].

Whether FH and LDL apheresis impact PTX3 levels is currently unknown. Therefore we assessed the effects of HELP LDL apheresis on plasma PTX3, selected pro-inflammatory cytokines and markers of oxidative stress in FH patients and their mutual associations.

## Materials and Methods

### Ethics Statement

The study was approved by the Ethical Committee of Verona University Hospital, and all participants gave written informed consent to it.

### Patients

19 heterozygous FH patients (M/F:11/8) undergoing regular HELP LDL apheresis every two weeks because of severe hypercholestereolemia inadequatelly controlled by maximal statin treatment for at least 6 months and previous cardiovascular events were recruited. 20 age- and sex- matched subjects were studied as control. Inclusion criteria were outpatients who were 18 years old or older. LDL elimination from plasma was achieved through HELP apheresis (LDL precipitation by acetic acid and heparin, Braun, Melsungen, Germany). Anticoagulation was performed with heparin (1000–5000 IU as a bolus and up to 3000 IU/h continuously). Exclusion criteria were the diagnosis of malignancies, chronic autoimmune, liver, thyroid and concomitant acute inflammatory diseases. Patient characteristics are shown in Table 1.

**Table 1:** Anthropometric and clinical characteristics of study subjects.

	FH	Control
n	19	20
Gender (M/F)	11/8	11/9
Age (years)	60±2	56±5
Body mass index (kg/m^2^)	27±1	27±3
SBP (mm Hg)	131±2	139±4
DBP (mm Hg)	80±2	83±6
Smoking status	3 current/16 never	2 current/18 never
Diabetes	1	-
Family history CAD	All	-
Previous cardiovascular events	All	-
LDL apheresis vintage (years)	7.8±1.6	-

FH: familial hypercholesterolemia. Data are means±SEM. SBP: systolic blood pressure; DBP: diastolic arterial pressure; CAD: coronary artery disease.

### Clinical data

On a baseline visit from each patient a detailed medical history and a physical examination were obtained. Then, in the morning of the scheduled day, FH patients were admitted to the Clinic for their regular HELP LDL apheresis. A blood sample was withdrawn immediately before and at the end of apheresis. In a subgroup of patients a time-course study was performed and additional blood samples were collected after 5, 10 and 14 days.

### Laboratory data

Plasma glucose, triglycerides, total, HDL and LDL cholesterol were assessed with standard techniques. Plasma fibrinogen was determined by the Clauss method.

Plasma TBARS were measured using a commercially available kit (Oxitek, Zeptometrix Co, Buffalo, NY) following the manufacturer's recommendations. Plasma high-sensitivity C-reactive protein and PTX3 were measured using ELISA kits (Diagnostics Biochem, London, Ontario, Canada; Perseus Proteomics, Tokyo, Japan intra-assay coefficient of variation: <4.1%, inter-assay coefficient of variation: <4.3%). TNFα was measured by using MILLIPLEX MAP Kit Human High Sensitivity Cytokine (EMD, Millipore, Billerica MA, USA); intra-assay coefficient of variation was <10.6%, inter-assay coefficient of variation: <9.8%. The minimum detectable concentration for TNFα was 0.07 pg/mL.

### Statistical analysis

Data are expressed as mean±SEM. ANOVA followed by Student's t test for paired or unpaired data were used as appropriate to compare variables between the experimental groups. Post-hoc analysis was performed, when appropriate, using Bonferroni's adjustment. Multivariate regression analysis was performed to adjust the relationship between PTX3 and apheresis vintage for age, gender and CRP. StatView software was used for statistical analysis. P values of 0.05 or less were considered statistically significant.

## Results

**Figure 1:**
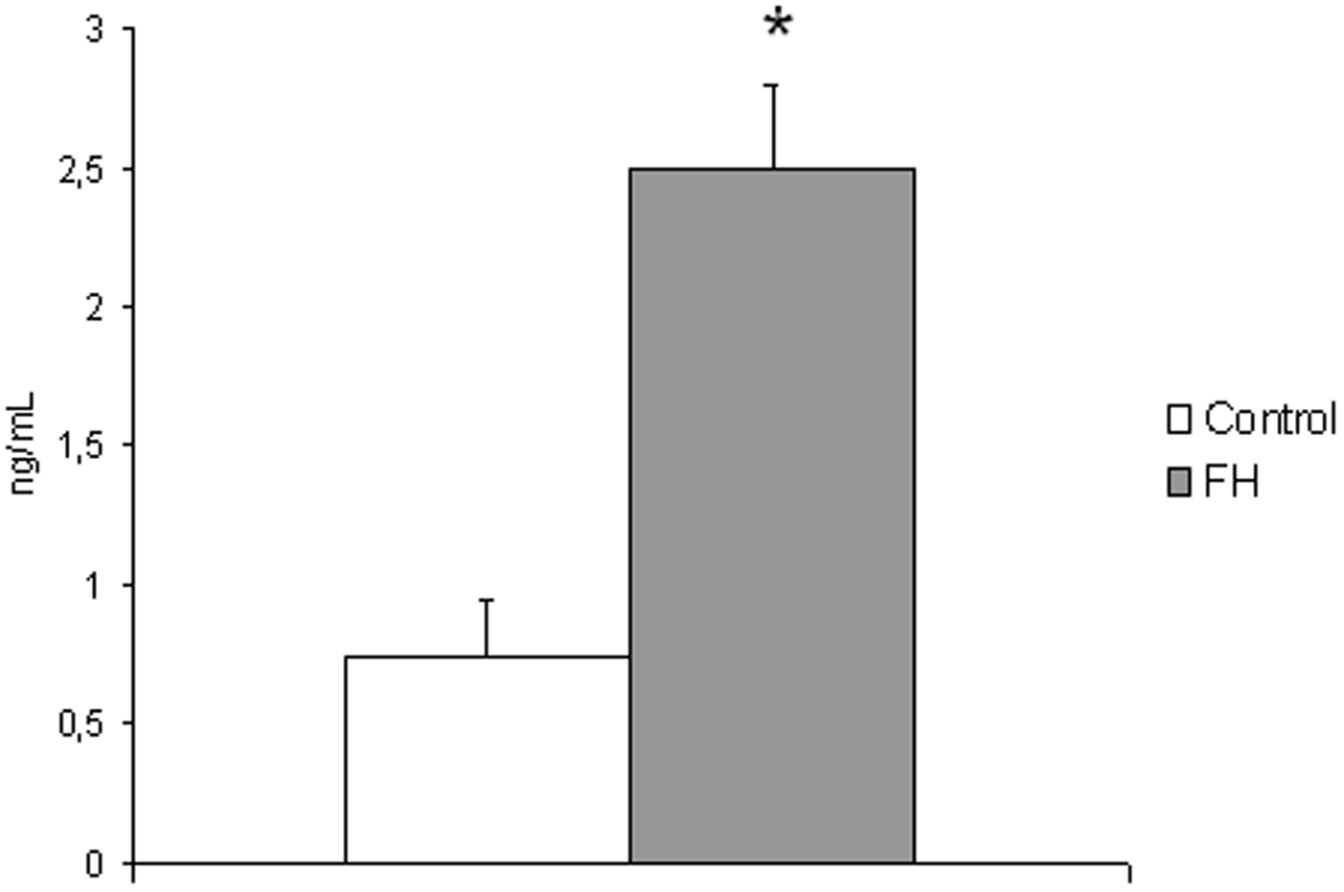
Plasma levels of PTX3 in control (n = 20) and FH patients (n = 19). P = 0.0002.

At baseline, FH patients had threefold higher (p = 0.0002) plasma PTX3 than matched control subjects (Fig.1). In the patients higher PTX3 was positively associated with increasing age (p = 0.001), female gender (p = 0.047) and higher CRP (p = 0.05) and negatively with HELP LDL apheresis vintage (p = 0.01) (Table 2). The latter association held true (p = 0.05) in multivariate modeling including CRP as well as age and gender (Table 3). In contrast, no correlation was detected with plasma lipids, TNFα and TBARS.

**Table 2:** Univariate analysis of plasma baseline PTX3 with anthropometric and laboratory parameters in FH patients.

	r	p
Gender (F/M)	0.46	0.047
Age (years)	0.69	0.001
Body mass index (kg/m^2^)	−0.41	0.08
SBP (mm Hg)	−0.39	0.3
DBP (mm Hg)	−0.49	0.17
Blood glucose (mg/dL)	−0.12	0.63
Duration of apheresis (years)	−0.63	0.01
Total cholesterol (mg/dL)	0.11	0.66
LDL cholesterol (mg/dL)	0.09	0.82
HDL cholesterol (mg/dL)	0.05	0.7
Triglycerides (mg/dL)	−0.05	0.8
Fibrinogen (mg/dL)	0.40	0.14
CRP (mg/L)	0.50	0.05
TNFα (pg/mL)	0.27	0.4
TBARS (nmol/mL)	0.11	0.8

FH: familial hypercholesterolemia. SBP: systolic blood pressure; DBP: diastolic arterial pressure; CRP: C reactive protein.

**Table 3:** Multiple regression model predicting LDL apheresis vintage in FH patients.

	Coefficient	P value
*FH patients (n = 19, r = 0.69)*		
Age (years)	0.18	0.35
Gender (female)	0.84	0.8
PTX3 (ng/mL)	−3.55	0.05

PTX3: pentraxin 3, CRP: C reactive protein.

**Figure 2:**
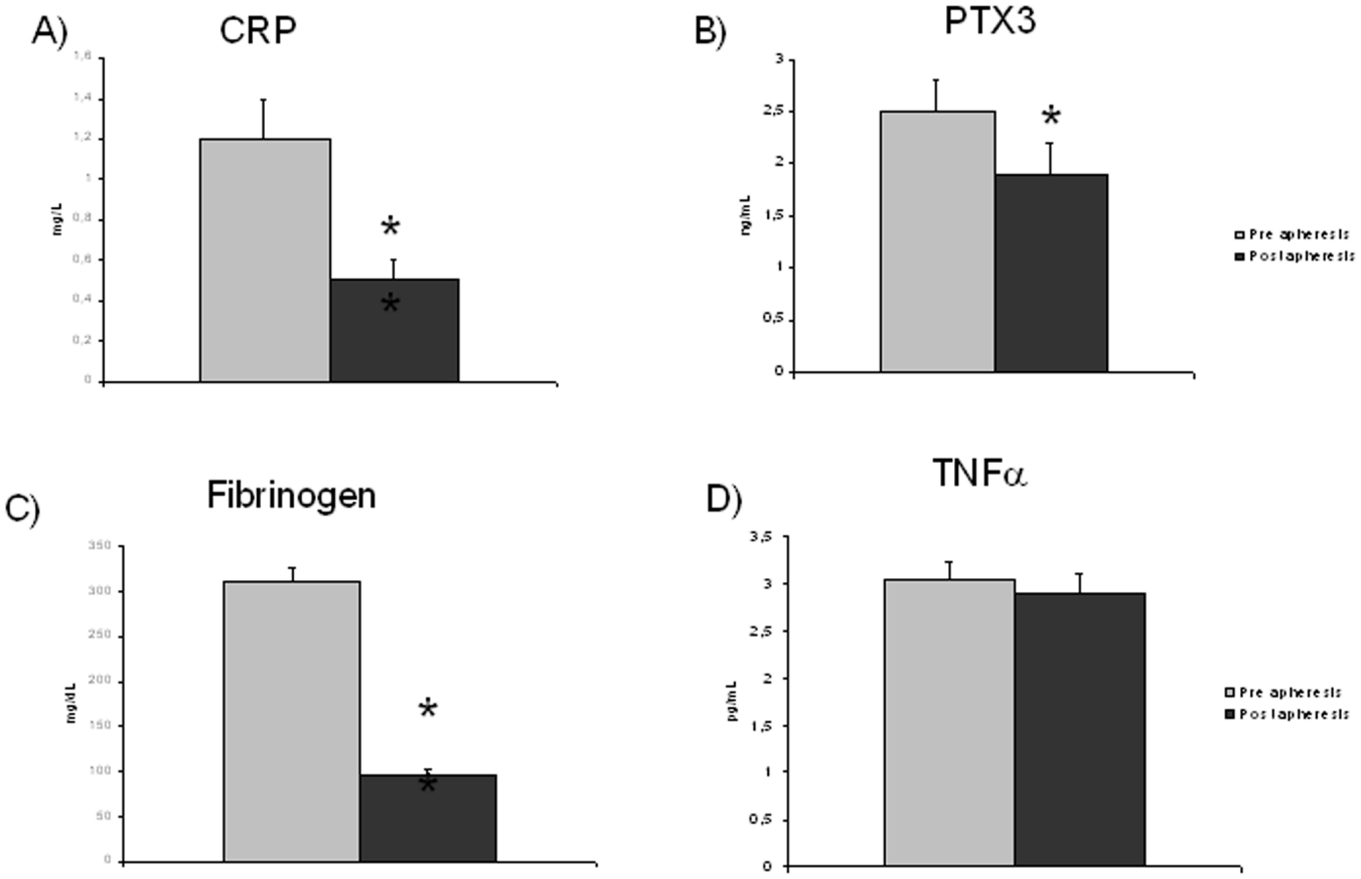
Plasma concentrations of inflammatory proteins A) CRP, B) PTX3, C) fibrinogen, D) TNFα in study patients. n = 19 both before and after HELP LDL-apheresis. *P≤0.04; **P≤0.003.

A single HELP LDL apheresis treatment acutely decreased (p≤0.0001) plasma total (from 268±11 to 125±7 mg/dL, −52±2%), LDL (from 176±10 to 65±6 mg/dL, −63±3%) and HDL cholesterol (from 52±4 to 45±4 mg/dL, −17±2%) as well as triglycerides (from 195±26 to 104±22 mg/dL, −50±4%), as expected. Substantial reductions in acute phase proteins CRP, fibrinogen and PTX3 were also observed by respectively 55, 65, and 20% (p≤0.04) (Fig. 2) as well as in the oxidative stress marker TBARS (by 19%, from 1.7±0.1 to 1.4±0.1 mmol/mL, p = 0.02). In contrast, TNFα did not decrease following HELP LDL apheresis (Fig. 2).

**Figure 3:**
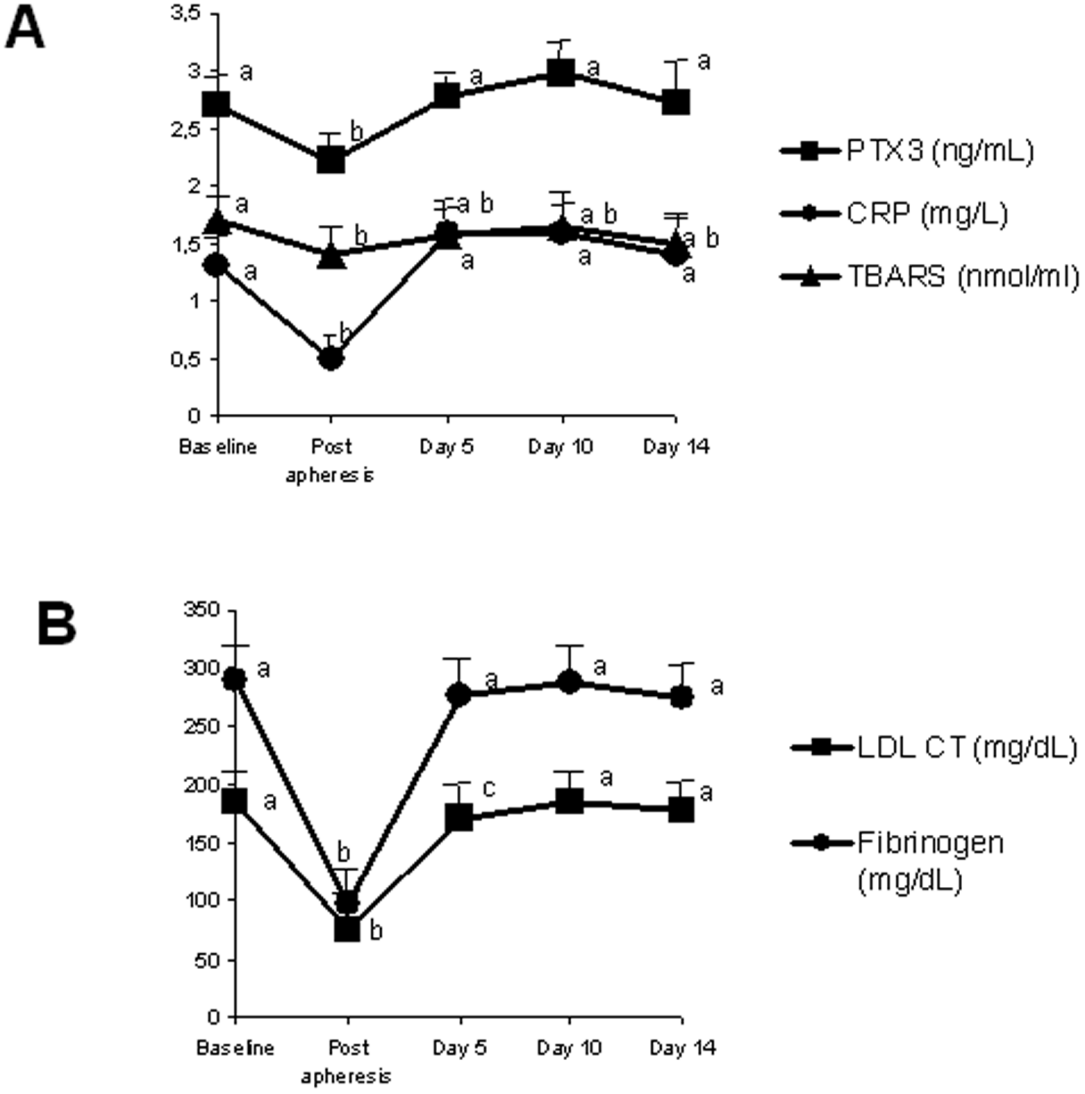
Fourteen-days trend analysis of pre- and post-treatment A) PTX3, CRP and TBARS plasma levels and B) LDL-cholesterol (LDL-CT) and fibrinogen in nine patients on chronic HELP LDL-apheresis. Within each curve, values sharing the same superscript letter do not differ significantly (P<0.05).

A time-course experiment in a subgroup of 9 patients demonstrated that within 14 days all parameters rebounded to pre-treatment values: plasma LDL, fibrinogen, CRP, TBARS and PTX3 (Fig. 3). In univariate analysis, no association was detected between HELP LDL apheresis-induced mean decrease in PTX3 and CRP and mean reduction in LDL cholesterol (PTX3: r = 0.38, p = 0.16; CRP: r = 0.48, p = 0.06).

## Discussion

This study provides the first evidence of the effects of FH and of HELP LDL apheresis on PTX3 plasma levels. FH patients on chronic statin and HELP LDL apheresis treatment show higher PTX3 levels than healthy subjects. HELP LDL apheresis acutely determines a rapid decrease of PTX3, which rebounds to pretreatment levels within 5 days after treatment. In addition, a negative association between PTX3 and apheresis vintage was observed.

Higher plasma PTX3 represent a consistent finding in this group of FH patients. Mean concentrations of PTX3 were over threefold higher in FH than those measured in control subjects, in spite of concomitant maximal statin treatment, which significantly reduces PTX3 concentrations [11], [25]. Indeed, measured PTX3 values in both groups are in line with those documented in subjects with cardiovascular risk factors including hypercholesterolemia [11], [26]. Statins are routinely administered to reduce LDL cholesterol and vascular inflammation in FH. When they fail to achieve LDL cholesterol target levels, LDL apheresis is considered [13], [14]. Extracorporeal treatment as in HELP is hampered by blood-biomaterial interaction, which may trigger inflammation; however acute adverse events during the procedure are rare [15]. In this study PTX3, a specific and sensitive marker of vascular activation decreased by about 20% following HELP LDL apheresis. The underlying mechanisms are unclear; either low buffer pH or heparin used in HELP may precipitate PTX3 along with several other molecules which are non-selectively removed during the procedure [27]. Since percent reduction of LDL cholesterol, triglycerides and fibrinogen was similar to previous data [18], [23], [28] and since plasma albumin did not differ before and after each session (data not shown), as expected [28], it is unlikely that dilution is responsible for PTX3 reduction in HELP apheresis. Based on these observations it is currently unknown whether acute PTX3 decrease is HELP-specific or other apheresis systems share the same effect.

LDL apheresis is the most efficient approach to achieve a rapid drop not only in plasma LDL but also in several procoagulatory, prooxidative and proinflammatory mediators, including fibrinogen, plasminogen, oxidative stress markers, adhesion molecules and CRP [17]. In addition, a single session has been demonstrated to increase coronary artery blood flow and luminal diameter [16]. For all these reasons LDL apheresis has been proposed as a potential therapeutic strategy in acute coronary syndromes [20]. In this setting however acute elevations of PTX3 may represent a protective, counteractive response to vascular injury and lowering PTX3 may be detrimental to clinical outcome [12]. Therefore the overall effect of HELP LDL apheresis in the acute setting is currently unclear.

TNFα is a known stimulator of PTX3 expression [6], [7] and variable effects on its plasma levels have been described during LDL apheresis [17], [29]. In this study however no correlation was found between baseline and post-apheresis PTX3 and TNFα, and unlike PTX3, TNFα did not decrease following HELP LDL apheresis. Likewise, no correlation was found between PTX3 and plasma HDL, which have been reported to induce PTX3 expression in endothelial cells [30]. In contrast with previous studies [8], [26], we did detect an association between baseline PTX3 and CRP. The reason for the discrepancy is not fully understood. One hypothesis is that the peculiar clinical characteristics of our patients (FH) can account for the finding. As such, a previous study confirmed an association between PTX3, CRP, LDL cholesterol and coronary artery disease severity in patients with intermediate-high atherosclerosis severity [31].

Chronic LDL apheresis is extremely effective in reducing the burden of atherosclerosis in FH [32], [21] as a result of modified lipid, proinflammatory and procoagulant profile despite rebounding to pretreatment levels after each session. Serum PTX3 is acutely elevated in critically ill and chronic cardiovascular patients [10], [33] correlating with clinical scores that reflect disease extension and complexity [31], [33]. The current data confirm these observations, as higher PTX3 was observed in subjects with shorter duration of treatment; this inverse relationship was independent from other clinical factors known to influence PTX3 levels, namely age and gender [34]. Lowering PTX3 level in high risk patients is associated with disease regression and reduced incidence of cardiovascular events [11], [35]; whether reduced PTX3 in patients with longer duration of treatment is a direct or indirect effect of HELP LDL apheresis remains to be elucidated.

In this study overall reduction rates of LDL cholesterol, triglycerides, fibrinogen and CRP were similar to results from previous studies [18], [22], [23]. However, mean reductions in plasma total and LDL cholesterol induced by HELP LDL apheresis were not related with the decrease in plasma PTX3 and CRP, indicating that plasma lipid pattern is not a major drive in the LDL-apheresis related changes of the inflammatory proteins PTX3 and CRP.

Our study has some limitations. First, direct assessment of atherosclerosis severity was not available at the time of the study, although a positive relationship between the extension of vascular disease and PTX3 levels in subjects with cardiovascular risk factors is well established [31], [33]. Second, due to small variations in PTX3 concentrations over time, a further sample collection for trend analysis of PTX3 levels was not acquirable within the study period. Despite these observations, the negative association between PTX3 and HELP LDL apheresis vintage suggests that chronic intensive lipid-lowering with HELP LDL apheresis has beneficial effects in patients with extensive cardiovascular disease.
